# 3D Non-Local Neural Network: A Non-Invasive Biomarker for Immunotherapy Treatment Outcome Prediction. Case-Study: Metastatic Urothelial Carcinoma

**DOI:** 10.3390/jimaging6120133

**Published:** 2020-12-03

**Authors:** Francesco Rundo, Giuseppe Luigi Banna, Luca Prezzavento, Francesca Trenta, Sabrina Conoci, Sebastiano Battiato

**Affiliations:** 1 STMicroelectronics—ADG Central R&D Division, 95125 Catania, Italy; 2 Medical Oncology Department, United Lincolnshire NHS Hospital Trust, Lincoln LN2, Lincolnshire, UK; giuseppe.banna@nhs.net; 3 DIEEI, University of Catania, 95125 Catania, Italy; lucabprezzavento@gmail.com; 4 IPLAB, University of Catania, 95125 Catania, Italy; francesca.trenta@unict.it (F.T.); battiato@dmi.unict.it (S.B.); 5 Department of Chemical, Biological, Pharmaceutical and Environmental Sciences, University of Messina, 98100 Messina, Italy; sconoci@unime.it

**Keywords:** 3D-CNN, immunotherapy, radiomics, self-attention

## Abstract

Immunotherapy is regarded as one of the most significant breakthroughs in cancer treatment. Unfortunately, only a small percentage of patients respond properly to the treatment. Moreover, to date, there are no efficient bio-markers able to early discriminate the patients eligible for this treatment. In order to help overcome these limitations, an innovative non-invasive deep pipeline, integrating Computed Tomography (CT) imaging, is investigated for the prediction of a response to immunotherapy treatment. We report preliminary results collected as part of a case study in which we validated the implemented method on a clinical dataset of patients affected by Metastatic Urothelial Carcinoma. The proposed pipeline aims to discriminate patients with high chances of response from those with disease progression. Specifically, the authors propose ad-hoc 3D Deep Networks integrating Self-Attention mechanisms in order to estimate the immunotherapy treatment response from CT-scan images and such hemato-chemical data of the patients. The performance evaluation (average accuracy close to 92%) confirms the effectiveness of the proposed approach as an immunotherapy treatment response biomarker.

## 1. Introduction

Urothelial carcinoma (also known as bladder cancer) is the most common histological type of urinary tract carcinoma. It accounts for about 3% of all cancers [1]. It is more common between the ages of 60 and 70, which is three times more frequent in men than in women and it is associated with about 165,000 global deaths every year and a five-year survival of approximately 5% in the advanced stage [1]. Metastatic Urothelial Carcinoma (mUC) occurs at disease onset in approximately 10% of patients, mostly arising from the evolution of previous superficial or infiltrating forms [2]. The current standard first-line treatment of mUC is platinum-based chemotherapy. In terms of progression-free survival (PFS), an improvement has been recently reported with the combination of chemotherapy and immunotherapy as a first-line treatment in mUC [3]. However, a significant and mature overall survival (OS) data are still expected. The median OS with cisplatin-based regimens varies between 12 and 15 months [3], while it is approximately 9 months in patients not eligible for cisplatin treatment because of the severity of the side effects treated with carboplatin-based regimens [4]. Immunotherapy has become the standard second-line treatment of mUC based on two phase III studies with Immune Checkpoint Inhibitors (ICIs) immunotherapeutic drugs such as Atezolizumab and Pembrolizumab with a median OS reported of 8.6 [5] and 10.3 months [6], respectively. These results have recently been confirmed in a large population with Atezolizumab [7]. ICIs are also a treatment option in first-line therapy of patients with non-cisplatin-based mUC based on phase Ib-II studies with a median OS between 8.7 and 18.2 months [8,9,10,11,12,13,14]. However, no more than about 20% and 30% of patients have a disease response with ICI in post-platinum and first-line treatment, respectively, even though these responses tend to be more durable than those obtained with chemotherapy [12,13,14]. Therefore, it is a priority to identify and select those patients who can really benefit from immunotherapy, even though, at the moment, there are still no reliable and clinically available biomarkers to properly choose patients who respond or progress with ICIs [15,16,17,18]. Since the activity of a high tumor mutational burden and of infiltrating lymphocytes has been associated with a higher probability of response to immunotherapy [8,19], some researchers have tried to characterize the tumor environment by integrating data from instrumental imaging and testing a reliable correlation with patients’ outcome, paving the way to the emerging field of radiomics [20]. Radiomics aims to extract a large number of quantitative features from high-throughput medical images by taking advantage of the recent data-characterization learning-based algorithms. Some studies show that these methods have the capability to uncover disease characteristics that, otherwise, cannot be identified by human observers [20]. In this paper, we propose an innovative deep learning pipeline used for the prediction of response to ICIs’ immunotherapeutic treatment for patients with advanced metastatic bladder cancer (mUC) who have progressed following a first-line platinum-based chemotherapy. The architecture of the proposed deep model is based on 3D Densely connected Convolutional Neural Network (3D-DCNN) with separable convolutions and self-attention mechanisms through non-local blocks [21]. The model processes computed tomography (CT-scan) imaging data and discriminates patients with high chances of response (complete, partial response, or, at least, stable disease), from those that, instead, are likely to show disease progression. 3D-DCNNs have been widely used in medical imaging for segmentation applications [22] as well as for cancer lesion characterization [23]. We leverage the success of these models and extend them with a self-attention mechanism, based on non-local blocks, for better learning long-range dependencies among the input data (segmented CT scans cancer lesions). As mentioned, it is, thereby, verified that all the patients have undergone a histological exam that confirmed the presence of bladder cancer. Experimental results, carried out on a dataset consisting of 41 patients with bladder cancer (which include, as a whole, 106 cancer lesions to be analyzed), show that the devised self-attention-based model leads to a better characterization of the bladder cancer (i.e., the associated feature maps) and of the radiological visual features for predicting treatment outcome with respect to the state-of-the-art methods. The paper is organized as follows. “Related Works” reviews state-of-the-art pipelines with a focus on deep learning models in the medical imaging field. The section “Methods and Materials” provides an accurate description of the proposed pipeline, together with details about the adopted training and validation dataset procedures. Experimental results of the proposed method as well as a comparison to state-of-the-art models are given in the “Results and Discussion” section. Finally, in the “Conclusions” section, the implications of the proposed approach are discussed, and some ideas for future extensions are briefly outlined.

## 2. Related Works

The feasibility of predicting the response to immunotherapy treatment for patients with neoplastic diseases in the metastatic phase has been recently investigated using standard machine learning and deep learning methods. Traditional machine learning methods based on the analysis of high-dimensional clinical data and CT-based diagnostic imaging have been proposed in order to predict the outcome of bladder cancer treatments [24,25,26]. Specifically, Reference [24] reports a comparative analysis of different machine learning methods used to process high-dimensional clinical data with the aim to predict mortality after a radical cystectomy in a large dataset of bladder cancer patients. Among the analyzed methods, the Regularized Extreme Learning Machine yields the highest average accuracy. In Reference [25], the authors analyzed the performance of multiple machine learning methods applied to process CT urography imaging data of each subject belonging to the recruited dataset of 84 patients, namely, Linear Discriminant Analysis (LDA), Neural Networks (NNs), Support Vector Machine (SVM), and a Random Forest (RAF) Classifier with SVM slightly outperforming the others. Analogously, Reference [26] compares multiple traditional learning methods (e.g., SVM, Bagged SVM, K-nearest neighbor, AdaBoost, Random Forest, and Gradient Boosted Trees) to automatically determine disease status and prognosis of patients with bladder cancer while also suggesting a recommendation treatment, i.e., neoadjuvant, definitive, and adjuvant therapy. All methods showed a fair accuracy (in terms of sensitivity and specificity), demonstrating the suitability of machine learning in addressing oncological applications. Despite the above machine learning methods showing promising performance in supporting physicians in their clinical investigation, they still rely on an old-fashioned approach, i.e., feature engineering followed by learning methods. With the rediscovery of deep learning in 2011 and the availability of large computation resources (thanks to GPU), the learning paradigm has radically changed in many tasks from computer vision to speech analysis to medical image analysis. About mUC investigation, the Deep Learning breakthrough has solicited the medical image research community to investigate advanced methods mainly for bladder cancer segmentation [27,28,29,30], while little effort has been put forward for studying bladder tumor chemotherapy efficacy [31,32]. For instance, in Reference [31], the authors investigate AlexNet [32] (with few architectural variants in terms of kernel size, padding, and stride) for assessing chemotherapy treatment efficacy of bladder cancer, starting from manually-segmented Computed Tomography (CT) scans. Experimental results showed an average accuracy of 86% (Sensitivity 90%, Specificity 89%) on immunotherapy treatment estimation. In Reference [33], the authors propose a two-stage deep-learning analysis pipeline for bladder cancer. The first stage deals with automated CT scan lesion segmentation (Auto-Initialized Cascaded Level Sets system) and the other one handles treatment response prediction (with an AUC of 0.73) using the previously segmented lesions. As for immunotherapy, some of the authors of this paper in Reference [34] proposed a deep network based on auto-encoders for cancer treatment outcome prediction in patients treated with Pembrolizumab (anti PD-1/PD-L1 ICIs checkpoint). To our knowledge, Reference [34] is one of the first works employing artificial intelligence for the prediction of immunotherapy outcomes. The work herein described extends the pipeline proposed in Reference [34] by introducing a 3D deep model enriched with a self-attention mechanism, which improve the learning phase of the joint visual and clinical data features. While it is important to have accurate automated computer methods for cancer treatment efficacy prediction, the interpretability and the explanation for why these methods reach specific decisions are of equal importance for the involved physicians. For these reasons, an ideal model should be accurate as well as explain what features it employs for its decisions. This need has been extensively outlined in both the artificial intelligence and medical imaging domains [35,36,37,38,39]. Our work contributes to the research area in automated immunotherapy outcome prediction through visual investigation of CT scans, as follows.

- We present a generalizable deep model that combines 3D densely connected convolutional layers empowered with self-attention mechanisms for estimating automatically the efficacy of bladder cancer immunotherapy treatment, purely based on CT imaging analysis.
- We investigate, through interpretability methods, such as Grad-CAM [40], what are the radiological CT visual features that most likely act as biomarkers for immunotherapy treatment outcome, thus, providing a potentially invaluable support to medical staff in evaluating the progress of bladder cancer. To the best of our knowledge, to date, no method has tackled the task herein proposed, from both the automated treatment outcome prediction and interpretability perspectives.

## 3. Materials and Methods

**Figure 1:**
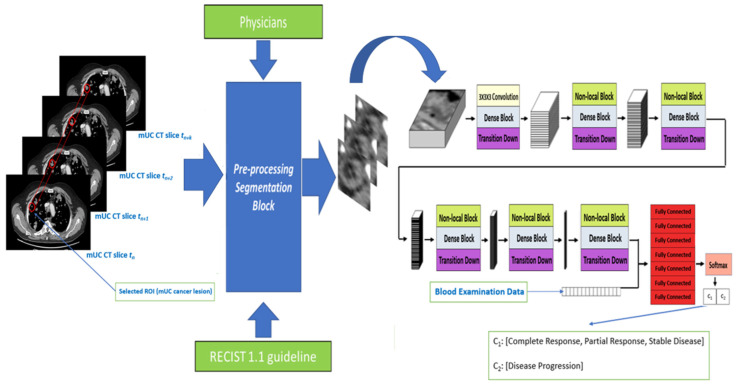
The proposed 3D Densely connected Convolutional Neural Network (DCNN) with Non-Local blocks architecture: Input data is a segmented lesion (ROI on CT scan) traverses the deep network (a sequence of dense and non-local blocks) and it is then classified as belonging to class C1 or class C2.

In this section, we propose our deep learning-based approach for radiomics applied to CT-scan cancer imaging for estimating the outcome of the ICIs based immunotherapy treatment in patients affected by mUC. The proposed framework consists of a combination of 3D Densely Connected Convolutional layers (3D-DCNN) with non-local blocks [21], implementing a self-attention strategy to improve characterization of spatio-temporal dependencies of the neoplastic lesions in CT slices. The use of 3D deep convolutional layers is motivated by the results achieved by our previous work [34] demonstrating the correlation between the biological aggressiveness of bladder tumors with the dynamic morphological evolution of the interested CT lesions. Thus, a 3D deep network provides a very useful tool to characterize not only the static-spatial 2D morphology of the lesions, stratified in the chest-abdomen CT slices, but also the functional dependence between their 3D volumes and the immunotherapy treatment responsiveness [34]. However, as pointed out in bladder treatment cancer guidelines [41], not all the metastatic lesions (generated by the primary neoplasm) play a role in the analysis of the progression of oncological disease. For this reason, a set of guidelines known as Response Evaluation Criteria in Solid Tumors (RECIST) have been developed by the scientific community [34,41]. In this work, a detailed analysis of only the lesions compliant to the RECIST criteria is to be carried out [41]. Thus, to address these indications, we extend 3D convolution architectures with an implicit “attention” to make the models focus—through visual feature learning—only on the most significant parts of the RECIST lesion and their possible correlation. More specifically, the attention mechanism is implemented by concatenating non-local blocks [21] at different layers for capturing long-range dependencies at different scales. We report the problem of treatment outcome estimation as a binary classification task, i.e., the proposed model provides as output of two class probabilities including one in the case of complete/partial regression or stable disease (C1), and the other one for disease progression (C2). The flowchart of the whole approach is shown in Figure 1. The backbone of the proposed model is a sequence of dense blocks, similar to DenseNet Cha et al., 2016, but replacing 2D convolutions with 3D ones. The model processes as input a batch of 16 × 64 × 64 volume (16 slices, among the 64 available, each with a spatial resolution of 64 × 64) extracted from CT data and containing the RECIST 1.1 compliant lesion. This input data is first fed to a 3D convolutional layer with a kernel size of 3 × 3 × 3, providing an output of 32 features of depth. These feature maps will be processed by six dense blocks composed by [6, 8, 8, 8, 8, 6] 3D layers, respectively, with the same kernel size as the input layer, followed by ReLU non-linear activations. Each dense block is preceded by [0, 1, 2, 3, 4, 5] Embedded Gaussian Non-Local blocks [21], respectively, and each dense block is followed by a transition-down layer with a 2 × 2 × 2 max pooling. Thus, the input volume is processed by the described blocks (both dense and non-local) generating the feature maps, which will gradually decrease (in dimension) to a one-dimensional feature map. This feature vector is concatenated to additional non-visual features, i.e., blood-stream hemato-chemical indicators. The resulting feature map (size 751 × 1) then traverses six fully connected (FC) layers followed by RELU, except the last one that, instead, uses a SoftMax layer for the final binary classification. Negative log-likelihood loss is used during model training.

(1) $$Loss=\;-\overset{N}{\underset{i=1}{\sum }}X_{i}\log\left(P\left(X_{i}\right)\right)+\left(1-X_{i}\right)log\left(1-P\left(X_{i}\right)\right)$$

With *X_i_*, we setup the correct class label, while *P* (*X_i_*) represents the model’s predicted probability for the correct class. In the next section, more information on the architecture is highlighted, while additional details of the proposed deep model are given in Table 1.

A visual description of the deep architecture explained in Table 1 is shown in the graph of the model included in Figure 1. Specifically, starting from the pre-processed CT lesions fed as input, the convolutional blocks are highlighted, stacked with dense blocks and the transition layers whose generated feature maps transverse further processing blocks composed of non-local block stacked with a dense block and transition layer up to the final stack of fully connected and SoftMax from which the classification output will be generated. More details in the next sections.

### 3.1. Dense Blocks

The proposed 3D-DCNN includes densely connected blocks (dense blocks) with 3D separable convolution layers (both depth-wise and point-wise). Separable convolutions are adopted to improve efficiency (through a significant reduction of a model’s parameters), while not affecting the output performance. Each dense block consists of a sequence of dense layers, including a batch normalization layer, a 3D convolutional layer with a kernel size of 3 × 3 × 3 (depth-wise and point-wise separable) followed by a ReLU. Each dense block is followed by a transition down layer, aiming to half feature map dimension, and composed by a convolutional layer with a kernel size of 1 × 1 × 1 followed by a max pooling layer of kernel 2 × 2 × 2. The output of dense blocks is then passed to non-local blocks.

### 3.2. Self-Attention through Non-Local Blocks

Non-local blocks have been recently introduced [21], as a very promising approach for capturing space-time long-range dependencies and correlation on feature maps, resulting in a sort of “self-attention” mechanism. Non-local blocks take inspiration from the non-local means method, extensively applied in computer vision, and have demonstrated to significantly improve the performance of deep models [21]. Self-attention through non-local blocks aims to enforce the model to extract correlation among feature maps by weighting the averaged sum of the features at all possible positions in the generated feature maps [21]. In our pipeline, non-local blocks operate on almost each convolution layer to extract a feature in dependencies at multiple abstract levels for a holistic morphological modeling of the input RECIST lesions. The mathematical formulation of non-local operation is the following. Given a generic deep network as well as a general input data *x*, the employed non-local operation computes the corresponding response *y_i_* (of the given Deep architecture) at a *i* location in the input data as a weighted sum of the input data at all positions *j* ≠ *i*.

(2) $$y_{i}=\;\frac{1}{\psi \left(x\right)}\underset{\forall j}{\sum }\zeta (x_{i},\;\;x_{j})\beta \left(x_{j}\right)$$

With *ζ*(∙) being a pairwise potential describing the affinity or relationship between data positions at index *i* and *j*, respectively. *β*(∙) is, instead, a unary potential modulating ζ according to the input data. The sum is then normalized by a factor *ψ*(*x*). The parameters of *ζ*, *β*, and *ψ* potentials are learned during the model’s training and are defined as follows.

(3) $$\zeta (x_{i},\;x_{j})\;=\;e^{\Theta {\left(x_{i}\right)}^{T}\;\Phi (x_{j)}\;}$$

where *Θ* and *Φ* are two linear transformations of the input data *x* with learnable weights $W_{\Theta }$ and $W_{\Phi }$.

$$\Theta \left(x_{i}\right)=W_{\Theta }x_{i\;}$$

(4) $$\Phi \left(x_{j}\right)=W_{\Phi }x_{j\;}$$

$$\beta \left(x_{j}\right)=\;W_{\beta }x_{j}$$

For the *β*(∙) function, a common linear embedding (classical 1 × 1 × 1 convolution) with learnable weights *Wβ* is employed. The normalization function *ψ* is:

(5) $$\psi \left(x\right)=\;\sum_{\forall j}\zeta \left(x_{i},\;x_{j}\right)$$

In Equations (2)–(5), an Embedded Gaussian setup is reported [21]. The selection of the Embedded Gaussian based affinity function is compliant with recent self-attention approaches [21,42], specifically recommended for 2D or 3D applications.

### 3.3. Classification Layer: The Stack of Fully Connected

Once features are extracted through the combination of dense and non-local blocks, we obtain a one-dimension visual embedding (736 × 1). These features are then concatenated to an additional side of information consisting of blood tests and other clinical data. The combination of the two sets of features is then fed to a stack of fully connected (FC) layers. The objective of this FC stack is to find additional correlations among the aggregated (at different abstract levels) deep features and clinical data in order to enhance accuracy in assessing the ICI immunotherapy treatment outcome. The proposed full FC stack is composed by seven FC layers, with, respectively, 375, 187, 93, 46, 46, 46, and 2 neurons for each layer. In particular, the sequence of FC layers (whose optimal number of layers was decided during experimental results) is designed to create a hybrid visual-clinical features hierarchy to balance model complexity with learnability.

### 3.4. Dataset: Recruitment and Data Pre-Processing

**Figure 2:**
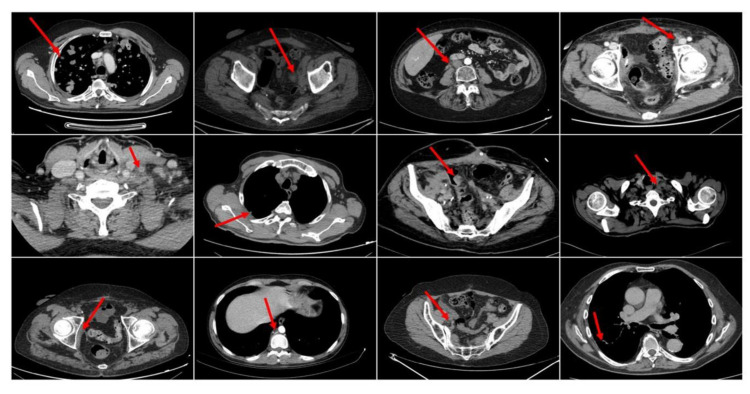
Some instances of the selected RECIST (Response Evaluation Criteria in Solid Tumors) compliant cancer lesions in Computerized Tomography (CT) imaging. The red arrow identifies an instance of the selected RECIST 1.1. compliant lesion.

We recruited a dataset of 43 CT/MRI scans from patients as part of a clinical study performed at a local hospital facility. The recruited patients (details are in Table 2) have histologically confirmed bladder cancer (mUC) progressing after a platinum-based chemotherapy and treated with an anti PD-L1 ICIs agent in the second or beyond line setting. All patients provided their written informed consent to the participation of clinical trials (Nr. D4191C00068 and MO29983 including the use of their clinical information for analysis approved by the Institutional review board (IRB) “Comitato Etico Catania 1”, Catania, Italy). The contribution, however, refers to the analysis of CT images for which the dataset will be limited to 41 patients who received an abdominal-chest CT discarding the other two who, instead, received MRI-based imaging. For each recruited patient, a chest-abdomen CT-scan was performed for cancer disease staging. The mentioned CT imaging was performed very close to the start of the ICIs immunotherapy treatment. Such instances of the collected chest-abdomen CT-scans are reported in Figure 2. The used imaging device consists of General Electronics (GE) CT scanner multi-slices (64 slices) with slice thickness of 2.5 mm. The working current is in the range of 10–700 mA. The working voltage is 120 kV and the pitch is 0.98 mm. As known, in a CT scanner, multi-slices of the spatial resolution in the scan plane is influenced by the convolution filter used to reconstruct the image by any other applied post-processing filter. It also depends on the number of projections that make up the image. This number, in turn, depends on the sampling rate and scan time. In this paper, we specify that the software of the previously mentioned GE tomograph allowed us to export CT scan slice images with each having a spatial resolution of 1440 × 810 pixels.

Each CT scan is complemented with the following clinical and personal history data (used in our learning model): primary tumor site, white blood cells (WBC), neutrophils, lymphocytes, eosinophils, platelets, albumin, Lactate DeHydrogenase (LDH), d-dimer, urine pH, proteins and Body Mass Index (BMI), age, gender, and tobacco use. The target of the proposed pipeline is closely related to the predictive estimate of the response to the ICIs immunotherapy treatment based on the analysis of the RECIST compliant lesion (often a metastases) identified by oncologists in the patient’s CT imaging. One of the most feared features of malignant cancer is their ability to metastasize to other parts of the body. Metastases can be spread through the blood and/or lymphatic route and clearly follow the anatomy of the interconnection of organs in the human body. The process by which oncologists define the cancer extension is called staging. With a special focus on bladder cancer, the staging requires the use of imaging (usually CT-scan and PET) to characterize the level of disease spread in the subject body [33,34]. For this reason, especially in the advanced mUC stages, CT scans show multiple lesions and radiologists/oncologists select the most significant ones (according to the RECIST guideline) for monitoring cancer evolution over time. The selection is carried out according to the previously mentioned RECIST guidelines that define inclusion criteria, CT scan procedure, patient assessment, lesion features, and how to monitor cancer over time. According to the RECIST 1.1 guideline, lesions of interest (target lesions) are those with the longest diameter (LD) in one dimension ≥20 mm (examples are shown in Figure 2). Lesion dimensions are used to set up a disease baseline for the patient’s assessment. According to RECIST 1.1, the sum of the longest diameter (LD) for all selected target lesions is the baseline LD. The baseline LD is used as a reference to evaluate the follow-up and treatment response of analyzed cancer. The LD value is then used for patient assessments after the oncological treatment. In particular:

- A patient shows a complete response (CR) to the medical treatment if all identified target lesions (LD sum) disappear at the end-treatment CT imaging.
- A patient shows a partial response (PR) to drug treatment if the target lesions (LD sum) are reduced by at least 30%.
- A patient shows a progressive disease (PD) if the Longest Diameter(LD) sum increases by, at least, 20% of the LD (LD sum, in case of multiple target lesions).
- A patient instead reports stable disease (SD) if no significant increase or decrease is observed on the target lesions.

The 41 patients included in this work were categorized, according to RECIST 1.1. All the CT RECIST 1.1 compliant lesions have been collected for a total amount of 106 RECIST 1.1 findings. Therefore, each RECIST 1.1. compliant lesion was treated individually even if it belonged to the same patient. These are some statistics of the collected clinical dataset. Furthermore, 30% of the patients were under the age of 60.91% of patients were male, with the remaining 9% female. A total of 33% of subjects had lymph node metastases, while the remaining 67% had various visceral metastatic lesions. Forty-three cases (target lesions) are referred to a complete/partial response or a disease stabilization following immunotherapy treatment (CR/PR/SD: Class 1), while 63 lesions are regarded to the disease progression despite anti-PD-L1 drug treatment (PD: Class 2).

### 3.5. Data Annotation, Training Procedure, and Evaluation Metrics

Data annotation was carried out by an expert oncologist. In particular, starting from the whole CT scan, the oncologist manually selected, according to RECIST 1.1 recommendation, all the target lesions to characterize the disease. 

Nowadays, all CT scanner imaging software allows the automatic selection of a Region Of Interest (ROI), according to certain spatial, dimensional, and/or morphological criteria. After this selection, a 64 × 64 bounding box area (ROI) around each selected lesion over 16 consecutive slices is extracted (thus, forming a 16 × 64 × 64 VOI i.e., the Volume of Interest). In order to ensure that the selection manually made by the oncologist includes the whole target lesion to be analyzed, ad-hoc ROI of spatial dimensions include the target lesion in its maximum extension (as it appears in the selected slices) that will be manually applied by the oncologist/physician. If the applied ROI dimension is different from the predetermined input size (64 × 64 in this case), a bi-cubic resizing of the ROI will be applied to bring it to the desired size, i.e., 64 × 64. The oncologist will select the lesion in a slice (the one in which there is the maximum extension of the lesion) and the latter will be propagated to all the selected input slices. The needed bi-cubic resizing will be extended to all the processed slices.

Once the VOI has been selected in the first slice, a software tool we have developed will automatically extract the same VOI in the other slices (for a total of 16) in order to characterize the morophological temporal dynamics of the lesion. As mentioned in the previous section, CT data was complemented with 15 additional clinical and hemato-chemical data that are converted into numeric representation and suitably normalized for being processed by the proposed model. These data are included in the *LabVector (i)* input as the index *i* is used to identify the patient. The temporal depth of processed CT slices (i.e., equal to 16) was identified as the one providing the best trade-off between performance and computational complexity, according to our previous work [34]. We noticed that, depending on the dimension, some RECIST compliant lesions do not appear visible in all 16 CT slices, and, in those cases, we zeroed (input data padding) the slices where the targeted lesion was not visible to keep the input temporal-depth fixed for our deep 3D model. The CT scans (better, VOIs of size 16 × 64 × 64) were properly labelled with reference to the two classes previously identified and described (C1 and C2). However, the selection of the samples in each dataset split was not performed randomly, but, in order to balance suitably, the presence of the patients of the two considered classes (C1 includes patients with some response to immunotherapy, i.e., CR/PR/SD cases, while C2 includes patients with a progressive disease (PD)), and, consequently, to ensure enough variability of the characteristics of the subjects. In particular, the dataset was configured as follows: 76 target lesions (28 of Class 1 and 48 of Class 2) were used for training and validation sessions, while the remaining 30 CT target image lesions (15 of Class 1 and 15 of Class 2) were used as a test set. Clearly, in configuring the test-set, we avoided using lesions of patients (although different) used in the training or validation set in order to improve the robustness of the validation process of the proposed pipeline. Unfortunately, immunotherapy treatment is still a relatively new strategy and the mechanisms of its functioning and interaction with the human immune system are not widely known and, as such, patients who do not respond to immunotherapy treatment are many more (currently) than those who respond positively to the ICIs’ stimulation and, therefore, building a dataset perfectly balanced between the two classes, which is not trivial [4,5,6,7,8,9,10,11,12]. In any case, to improve the validation reliability, we have implemented a cross-validation mechanism through a k-fold. Specifically, we cross-validated our deep model by configuring k = 5 and reporting the results of this procedure in Table 3 (mean and standard deviation for the main performance indexes). The output of our deep model for each input data (16 × 64 × 64 VOI with additional clinical data) is a (two) class probability vector on which, during training, we compute a negative log-likelihood loss with L2 regularization weighted by a factor λ = 0.0001. The mini-batch gradient descent was performed for minimizing the model loss, using the Adam optimizer, with an initial learning rate of 0.01, and a mini-batch size of 4. For data augmentation, we perform random translation and rotation (with a random degree value) along the spatial axis, consequently increasing the dataset dimension during the training session. Our deep model is implemented by using the Pytorch framework. Experiments were carried out on a server with 2 Intel Xeon E5620 CPU with 4 cores each, 96GB of RAM equipped with a Nvidia Quadro P6000 GPU with 24 Gbytes of video memory. In order to validate the performance of the proposed architecture with respect to other deep learning-based solutions, the following metrics have been used (FP: False Positive, FN: False Negative, TP: True Positive, TN: True Negative).

(6) $$Accuracy=\frac{TP+TN}{TP+TN+FN+FP},$$

(7) $$Sensitivity=\frac{TP}{TP+FN},$$

(8) $$Specificity=\frac{TN}{TN+FP},$$

(9) $$F1-Score=\frac{2\cdot TP}{2\cdot TP+FN+FP},$$

We considered a “True Positive,” which is the right classification of a patient who has shown a certain response to immunotherapy treatment (complete response (CR), partial (PR), or has a stable disease (SD)), and has been previously classified by our pipeline as belonging to class C1. Consequently, we will consider “True Negative,” which is a patient who previously classifies as belonging to class C2 and then correctly following the treatment does not show any response to the immunotherapy drug confirming a progression of the disease (PD). The “False Negative” and “False Positive” values are computed accordingly. The accuracy, sensitivity, specificity, and F1-score are evaluated—on the test-set during k-fold cross-validation so that they are reported as mean and standard deviations. The collected experimental results are discussed in the next section.

## 4. Results and Discussion

### Performance Analysis

In this section, we report the promising performance results of the proposed approach. As mentioned, the used dataset is composed by 41 patients (106 RECIST compliant input image lesions) recruited as part of a clinical trial including, for each patient, informed consent, chest-abdomen CT scan images, and their blood examinations collected before the start of treatment. Each patient was adequately labeled with histological confirmation of mUC. The cross-validation session is used to select the best model setup of the proposed architecture, i.e., when the maximum k-fold cross-validation accuracy is retrieved (3D deep architecture as reported in Figure 1, network layers structured as per Table 1, fixed learning rate of 0.01, and a weight decay of 0.00001, Adam optimization). As introduced, we compared our architecture with such state-of-the-art deep architectures in order to provide performance benchmarks regarding the proposed application. Specifically, in order to evaluate the improvement in terms of performance compared to similar 2D and 3D deep networks, the authors have validated the performance of the used DenseNet backbone having the same architecture of our pipeline (Table 1), but without the inclusion of Self-Attention (Non-Local Blocks) mechanisms and separable convolutions. In addition, the performance of the proposed method has been compared with respect to such classic architectures: ResNet-50, ResNet-101, VGG-19, and 3D extension of the classical DenseNet-201 [43]. Table 3 reports the collected experimental results and comparisons. The implemented 3D DenseNet backbone baseline (3D DenseNet) showed an accuracy of 0.640 ± 0.034 significantly lower than our full pipeline, which shows a higher accuracy equal to 0.922 ± 0.037. Additionally, in terms of sensitivity, specificity, and the F1-score, our architecture is significantly more performed (0.929 ± 0.053, 0.916 ± 0.047, and 0.922 ± 0.038, respectively) than the simple DenseNet backbone, thus, confirming the improvements that can be obtained in particular through the use of self-attention techniques and separable layers. Specifically, feature maps that suitably weight the spatiotemporal dependencies of the CT imaging selected target lesion (i.e., the result of non-local blocks application) provide more discriminative features to the FC stack. Moreover, the joint contribution of non-local blocks and separable convolution layers allows us to generate feature maps having an informative content that best characterizes the spatiotemporal dependencies between the CT imaging VOIs and treatment response of the associated patient. As well known, the usage of separable convolutional layers significantly reduces the risk of overfitting [30,31,32,33,34]. During this session, we also investigated the performance impact of the jointed link between hemato-chemical data *LabVector (i)* with visual features based on CT imaging. We, therefore, performed a testing session using the same proposed deep architectures but avoiding concatenating the data contained in the *LabVector (i)* (“3D DenseNet+NLB+SepConv” in Table 3). As hypothesized by the oncologists who followed the trial on which this study is based, there is a close correlation between the anamnesis and hematochemical data, the imaging data, and the patient’s response to immunotherapy treatment even though this correlation is not perfectly known to date. In fact, our tests revealed a considerable reduction in the overall performance of the tested deep networks if, as inputs, we only use CT visual imaging and do not integrate with blood and medical history (*LabVector (i)).* In more detail, the proposed architecture with visual input but without hemato-chemical data dropped in performance as it showed 0.878 ± 0.039 (Accuracy), 0.871 ± 0.054 (Sensitivity), 0.884 ± 0.075 (Specificity), and 0.877± 0.075 (F1-score) significantly lower with respect to the same proposed pipeline with hemato-chemical data (see Table 3). Therefore, the clinical data included in *LabVector (i)* play a significant role in the discrimination capability of the proposed pipeline so that it is worthy of further study. As reported in Table 3, the accuracy of the classical deep 2D architectures such as ResNet-50, ResNet-101, and VGG-19 is significantly lower than our approach, further confirming the promising performance of the proposed solution. We have compared our implemented pipeline with similar 3D architecture but with more layers (3D DenseNet-201). The performance of our architecture is significantly higher than the compared 3D deep classifier, as reported in Table 3. This confirms that the Self-Attention mechanisms realized through the inclusion of non-local blocks with embedded gaussian setup together with the separable layers allow us to obtain more discriminative and representative features maps (with respect to a deeper network as the 3D-DenseNet-201) of the correlation with the response to immunotherapy treatment. Evidently, the capability of non-local blocks to embed more precise spatial-temporal correlations in the analyzed CT lesions allows the generation of more discriminative feature maps than those obtained by increasing the convolutional layers in the deep classifier. This makes the proposed system particularly performing in the application herein described. All the networks analyzed for a comparison share the same input setup data. We discriminated some setups only in reference to our pipeline to demonstrate that the DenseNet backbone was necessarily enriched with the Separable Convolutional Layers as well as with hemato-chemical data and non-local blocks. The aim of the work is to analyze the impact of imaging in predicting the response to immunotherapy treatment. We tested our model with blood chemistry data only, obtaining poor performances with accuracy below 50% (in cross validation), clearly confirming that the contribution of imaging is fundamental for the overall performance of the proposed model.

**Figure 3:**
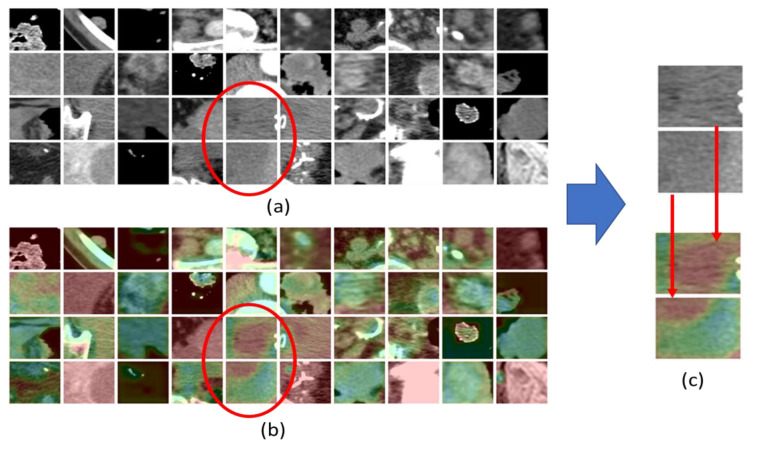
(**a**) RECIST (Response Evaluation Criteria in Solid Tumors) 1.1 compliant CT target lesions. (**b**) The corresponding Grad-CAM generated saliency maps. (**c**) A detail of the salient part of the processed RECIST lesion.

In any case, we remark that the proposed deep architecture aims not only to offer valid medical assistance to the physician but rather to highlight the most predictive visual patterns. In doing this, we have tried to investigate and adopt one of the most promising self-deep features explanatory methods already introduced in the scientific literature. The authors propose the usage of GradCAM introduced by Selvaraju et al., 2017 [40]. The GradCAM approach is intuitively very simple. It uses the gradient with respect to the generated convolutional features as a classification score in order to understand which parts of the input image are most significant for classification. By means of a simple combination between the activation saliency map generated by the GradCAM approach [40] with existing correlated input data, we are able to create a combined discriminative image pattern visualization easily understandable and able to guide the physician in the visual analysis of the imaging areas that have a greater weight in the discrimination/classification of the input dataset. More details are present in Reference [40]. Moving into our application, through GradCAM, we tried to understand which parts of the ROI extracted from the chest-abdomen CT images (containing the target RECIST compliant lesion), which were more significant for our 3D pipeline processing. Therefore, having obtained the corresponding GradCam based gradient-weighted activation maps, we combined them with the ROI of the target lesions extracted from the CT slices. The collected results we obtained are reported in Figure 3 and deserves further study in relation to such heuristically hypotheses made by several oncologists. Specifically, oncologists hypothesized that only certain parts of the RECIST compliant target CT lesions are significant in relation to the estimation of an immunotherapy outcome [34,44]. As evident from Figure 3, for some processed lesions (ROI), the GradCAM analysis highlighted such areas with greater salience (red area) than the others (green area). The salient visual areas of such input ROI-lesion are those that most contribute to the performance of the deep network, i.e., those that are best represented in the feature maps. This seems to confirm the hypothesis of some medical researchers.

The researchers hypothesized that the immune-cytochemical hyper-expression of the PD-L1 protein on tissue tumor sections or on cyto-block (together with the analysis of the Tumor Mutational Burder (TMB)) may be predictive of a positive response to the ICIs’ immunotherapy treatment [45]. This hyper-expression being found in the surface of such tumor cells is hypothesized to be evident in the morphology of the lesion of the cells of the primary tumor and likely of those of the related metastases [45]. Therefore, such researchers have hypothesized that only the areas of the lesions in which there is a high concentration of expression of the PD-L1 protein can be significant for the estimation of the response to the immunotherapy treatment [34,44,45]. Translating in our case, we investigated the concrete possibility that such micro-areas of the target lesions visible in the GradCam post-processed CT imaging, are significant for the estimation of the ICIs immunotherapy response of patients suffering from bladder cancer (assuming that the selected neoplastic target lesion shows such parts with great surface expression of the ligant protein PD-L1) [46,47]. From Figure 3, it is evident that, with reference to some target RECIST lesions identified in the chest-abdomen CT slice (Figure 3a), a specific sub-area of these lesions appear more significant (red) than others (green) and, therefore, are shown to have a greater weight in the estimation of the response to the immunotherapy treatment (Figure 3b). We have no scientific evidence that this saliency hyper-caption in the feature maps is related to the presence of cancerous cells with high expression of the PD-L1, but it is, however, significant that the explanation of the feature maps is indicative of a self-weighting of such image areas of a target metastatic lesion. Further investigations are underway on this interesting aspect.

## 5. Conclusions

In this paper, a problem of particular interest in the oncological field is tackled, namely the identification of a non-invasive and robust bio-marker that can assist the oncologists in the discrimination of patients potentially eligible for immunotherapy treatment. To the best of our knowledge, although there are several promising lines of research [4,5,6,7,8,9,10,11,12], there is no method that shows high accuracy in assessing patients who may undergo immunotherapy treatment from those who may not. However, many of the methods that are being studied are based on the research of the expression of the inhibitor ICIs PD-L1, which, however, is not highly discriminating and requires the invasive biopsy of the primary tumor [48,49]. For these reasons, we have investigated recent and innovative deep architectures in order to learn new patterns able to estimate the response to ICIs’ immunotherapy treatment based on imaging and clinical data of the cancer patient. Specifically, we investigated the problem of finding a non-invasive image-based biomarker for patients with metastatic bladder cancer. The architecture we have implemented and tested learns image-features from chest-abdomen CT imaging of the patients affected by mUC. As confirmed in Reference [44], a novel mechanism of action of ICIs’ treatment with immune and T cell activation leads to unusual patterns of response on CT imaging. For these reasons, an innovative 3D deep architecture with embedded self-attention mechanism and separable convolutions for hyper-parameters model optimization is implemented and analyzed. The proposed pipeline was tested on a sample of 41 recruited patients (clinical trial) for a total of 106 processed input visual RECIST 1.1. compliant lesions. The outcome of the proposed 3D Deep network is a preliminary estimation of the patients who respond or not to ICIs’ immunotherapy treatment, i.e., preliminary estimation of the patient belongs to Class C1 (i.e., subjects eligible as they have a high chance to show a complete or partial response or at least stabilization of the disease) with respect to patients belonging to Class C2 (patients who will manifest disease progression). As confirmed by experimental results reported in Table 3, the proposed 3D deep architecture shows very promising performance both in terms of accuracy as well as in terms of sensitivity and specificity, confirming that both adopted a self-attention mechanism. Separable convolutions significantly increase the classification ability of the deep model (as confirmed by a benchmark comparison with the baseline and classical backbones). The integration of blood hemato-chemical numerical data has further improved the classification performance of the proposed pipeline. We remark that the achieved promising results need to be confirmed in a bigger scale dataset (currently an extended clinical trial is under development). We are organizing a large-scale multicenter and multivariate clinical study that can validate with greater robustness. The promising results obtained from this contribution allows us to better understand the correlation between the morphological structure of the tumor lesion found in the CT diagnostic examination with the possible response to immunotherapeutic treatment and, therefore, with the iteration with the used drug.

Future works aim to investigate advanced methods for the automatic segmentation of RECIST target lesion in order to relief physicians from the burden to manually identify the lesion to be processed by our approach. Moreover, we are analyzing such methods based on usage of LSTM and Autoencoder-based architectures for modeling temporal dynamics of each pixel of the segmented CT lesion [50,51]. Interesting results have been collected integrating such bio-signals of the patients during the analysis of the visual pattern of the segmented CT lesion [52].

## 6. Patents

Francesco Rundo, Giuseppe Luigi Banna, Methods and System for Analyzing Skin Lesions, IT Patent Application Number 102016000121060, 29 November 2016—USA Patent Grant Number 10362985, 26 July 2019.

Francesco Rundo, Giuseppe Luigi Banna, Sabrina Conoci, Deep Learning Motion Algorithm for Lung Cancer Early Detection in Embedded Systems, IT Patent Application Number 102018000010833, 05 Dec 2018.

## Figures and Tables

**Table 1:** The layers specification of the proposed deep architecture.

Block	Output Size	Layer(s) Description	Layers Number
Convolution	32 × 16 × 64 × 64	3 × 3 × 3 convolution	1
Dense Block	128 × 16 × 64 × 64	Batch Normalization Rectified Linear Unit (ReLU) 3 × 3 × 3 depth-wise convolution 1 × 1 × 1 point-wise convolution	6
Transition layer	128 × 8 × 32 × 32	1 × 1 × 1 convolution, 2 × 2 × 2 maxpool	1
Dense Block	256 × 8 × 32 × 32	[…]	8
Transition layer	256 × 4 × 16 × 16	1 × 1 × 1 convolution, 2 × 2 × 2 maxpool	1
Dense Block	384 × 4 × 16 × 16	[…]	8
Transition layer	384 × 2 × 8 × 8	1 × 1 × 1 convolution, 2 × 2 × 2 maxpool	1
Dense Block	512 × 2 × 8 × 8	[…]	8
Transition layer	512 × 1 × 4 × 4	1 × 1 × 1 convolution, 2 × 2 × 2 maxpool	1
Dense Block	640 × 1 × 4 × 4	[…]	8
Transition layer	640 × 1 × 2 × 2	1 × 1 × 1 convolution, 2 × 2 × 2 maxpool	1
Dense Block	736 × 1 × 2 × 2	[…]	6
Transition layer	736 × 1 × 1 × 1	1 × 1 × 1 convolution, 2 × 2 × 2 maxpool	1
Concatenation	751	Integrates hematochemical patient’s data	1
Fully Connected	375	FC, ReLU	1
Fully Connected	187	FC, ReLU	1
Fully Connected	93	FC, ReLU	1
Fully Connected	46	FC, ReLU	1
Fully Connected	46	FC, ReLU	1
Fully Connected	46	FC, ReLU	1
Classification	2	FC, Softmax	1

**Table 2:** Some statistics of the used clinical dataset. CR: Complete Response; PR: Partial Response; SD: Stable Disease; PD: Progressive Disease.

Dataset Field Description	Number	%
**Age**		
≤60	13	30
>60	30	80
**Gender**		
Male	39	91
Female	4	9
**Tobacco Use**		
Never	5	12
Current	16	37
Former	22	51
**Therapy Line**		
2	38	88
≥3	5	12
**Primary Tumor Site**		
Upper urinary tract	4	9
Lower urinary tract	36	84
Both	3	7
**Metastases Site**		
Lymph-nodes only	14	33
Visceral	29	67
**Treatment Response**		
CR/PR/SD	16 (43 target lesions)	37
PD	27 (63 target lesions)	63
**Follow-up Median -Months-**		
CR/PR/SD/PD	13.4	11.1–15.6
**Follow-up Imaging**		
CT-scan	41	
MRI (Magnetic Resonance Imaging)	2	

**Table 3:** Experimental performance benchmarking (mean ± standard deviation).

Model	Accuracy	Sensitivity	Specificity	F1-Score
2D ResNet-50	0.620 ± 0.052	0.604 ± 0.0078	0.636 ± 0.061	0.613 ± 0.058
3D DenseNet + H	0.713 ± 0.047	0.711 ± 0.041	0.716 ± 0.064	0.713 ± 0.043
3D DenseNet + SepConv + H	0.733 ± 0.049	0.729 ± 0.069	0.738 ± 0.047	0.731 ± 0.054
3D DenseNet	0.640 ± 0.034	0.636 ± 0.034	0.644 ± 0.048	0.638 ± 0.032
3D DenseNet + NLB + SepConv	0.878 ± 0.039	0.871 ± 0.054	0.884 ± 0.075	0.877 ± 0.041
Proposed	**0.922 ± 0.037**	**0.929 ± 0.053**	**0.916 ± 0.047**	**0.922 ± 0.038**
2D ResNet-101	0.829 ± 0.043	0.822 ± 0.054	0.836 ± 0.061	0.828 ± 0.043
3D DenseNet-201	0.856 ± 0.033	0.871 ± 0.047	0.840 ± 0.055	0.858 ± 0.032
2D VGG-19	0.667 ± 0.033	0.662 ± 0.069	0.671 ± 0.059	0.664 ± 0.041
Previous [34]	0.861 ± 0.023	0.815 ± 0.011	0.883 ± 0.048	0.810 ± 0.037
